# Conjugate buoyant convective transport of nanofluids in an enclosed annular geometry

**DOI:** 10.1038/s41598-021-96456-8

**Published:** 2021-08-24

**Authors:** M. Sankar, N. Keerthi Reddy, Younghae Do

**Affiliations:** 1 Department of General Requirements, University of Technology and Applied Sciences - Ibri, 516 Ibri, Oman; 2 Department of Mathematics, School of Engineering, Presidency University, Bengaluru, 560064 India; 3 Department of Mathematics, KNU-Center for Nonlinear Dynamics, Kyungpook National University, Daegu, 41566 Republic of Korea

**Keywords:** Applied mathematics, Thermodynamics

## Abstract

A vertical annular configuration with differently heated cylindrical surfaces and horizontal adiabatic boundaries is systematically studied in view to their industrial applications. In this paper, we investigate the effects of conjugate buoyant heat transport in water based nanofluids with different nanoparticles such as alumina, titania or copper, and is filled in the enclosed annular gap. The annulus space is formed by a thick inner cylinder having a uniform high temperature, an exterior cylindrical tube with a constant lower temperature, and thermally insulated upper and lower surfaces. By investigating heat transport for broad spectrum of Rayleigh number, solid wall thickness, thermal conductivity ratio and nanoparticle volume fraction, we found that the influence of wall thickness on thermal dissipation rate along wall and interface greatly depends on conductivity ratio and vice-versa. In particular, we uncover that the choice of nanoparticle in a nanofluid and its concentration are key factors in enhancing the thermal transport along the interface. Specially, copper based nanofluids produces higher heat transport among other nanoparticles, and for the range of nanoparticle concentration chosen in this analysis, enhanced thermal dissipation along the interface has been detected as nanoparticle volume fraction is increased. Our results are applicable to choose nanofluids along with other critical parameters for the desired heat transport.

## Introduction

Buoyant thermal transport of conventional fluids as well as nanofluids (NFs) in various finite-sized geometries has been widely investigated through theoretical simulations and experimental visualizations. This is mainly due to the direct relevance of these geometries in many vital applications ranging from cooling of electronic components to safety measures of pertinent devices, e.g., nuclear reactors. In particular, amongst the finite-sized geometries, the annular space formed by two vertical co-axial cylindrical tubes with different heating of side boundaries and insulated horizontal surfaces is considered as a suitable model problem aptly describing the physical configuration in many applications. David and Thomas^1^ had made a pioneering attempt to numerically explore the buoyant convection in an upright annular space. They performed simulations for wider spectrum of parameter ranges and proposed heat transport correlations in different flow regimes. Later, Kumar and Kalam^2^ made numerical simulations of convective flow, thermal transport analysis and reported the discrepancies existing in the results of Davis and Thomas^1^, and suggested new correlations to predict the thermal transport rates. Convective flow of different liquids in an annular enclosure has been experimentally investigated by Prasad and Kulacki^3^ for three different aspect ratios and by fixing the ratio of outer to inner radius, known as radius ratio, $(\lambda =5.338)$. Few applications such as crystal growth processes require mechanisms to resist or dampen the convective flow to design the defect-free products and this can be achieved by employing magnetic field by considering different fluids^4–7^. Sankar and co-workers^8,9^ made detailed investigations to analyze the impacts of thermal sources on buoyant convective motion of air in a discretely heated porous and non-porous annulus and observed discrete heating could enhance thermal transport compared to complete heating of wall. Later, Wang et al.^10^ investigated transient buoyant flow in the same geometry and reported new correlations to predict the thermal transport rates. The size and positional influence of thermal sources on hydrodynamic stability has been numerically examined by Mebarek-Oudina^11^ in an upright annular domain. Recently, Husain and Siddiqui^12^ made an experimental analysis of unsteady buoyant convective flow of water in tall and narrow annular region, and also presented theoretical simulations through a commercial software package. It is worth to mention that the above investigations mainly addresses the buoyant flow and associated transport processes in a vertical annular chamber without taking account of wall thickness effect.

The addition of nano-sized particles (NPs) in conventional liquids could effectively enhance the thermal transport rates and is substantiated through the predictions made by many theoretical simulations and experimental observations^13–15^. Thermal transport analysis of different NFs in finite shaped geometries have also received a great amount of attraction due to the requirement of effective cooling of electronic equipments^16,17^. One of the earliest attempt to investigate the buoyant motion of Al$_2$O$_3$ NF in an annular geometry has been made by Abouali and Falahatpisheh^18^. They performed extensive numerical simulations by considering wide spectrum of parameter ranges and proposed thermal transport correlations for square and annular geometries. Cadena-de la Pe$\tilde{n}$a et al.^19^ conducted experiments to analyze cooling mechanisms of oil-based nanoliquids by considering two different NPs and found thermal transport enhancement with NFs. The impacts of discrete thermal sources of different lengths and locations on buoyant motion of NFs in an annular domain reveals interesting flow features and enhanced thermal transport as compared to uniform or complete heating^20,21^. Recently, Keerthi and Sankar^22^ presented numerical simulations to reveal the consequences of different non-uniform heating of annular boundaries on the convective motions of Cu-based NF and identified an appropriate heating condition to enhance the thermal dissipation rates. The convective motions of various NFs in horizontal and tilted annular configurations with and without fins have also been reported^23,24^.

Among the finite shaped enclosures, rectangular and square geometries have been widely used in analyzing the nanofluid buoyant motion by considering various constraints affecting the flow and thermal transport mechanisms. Khanafer et al.^25^ presented detailed analysis on thermal transport enrichment for Cu-based NFs in a square geometry and proposed a theoretical model and heat transport correlations to estimate nanofluid thermal performance. Later, this study was extended by Jou and Tzeng^26^ by taking NP dispersion into consideration and aspect ratio. Many investigations analyzed the buoyant motion and thermal dissipation of different NFs in two-dimensional plane geometries by considering various models for fluid properties and identified an appropriate model for thermal transport enhancement^27–29^. Oztop and Abu-Nada^30^ addressed the idea of choosing the type of nanofluid for flow behavior and thermal transport enhancement. The type of thermal boundary condition greatly influences the convective thermal transport characteristics which has been discussed in detail by Basak and Chamkha^31^. Through numerical predictions it has been observed that utilizing nanofluid in an enclosure predominantly improves convective heat transfer^32^. Roy^33^ examined nanofluid buoyant motion in the annular section between a square geometry and three distinct interior geometries, such as circular or elliptical or rectangular cylinder and found the inner shapes has profound impacts on thermal dissipation rates compared to a square geometry. Using combined Lagrangian and Eulerian modeling, Sharaf et al.^34^ investigated the convective motion and nanoparticle dissemination in a microchannel formed by parallel plates and brought out inaccuracies in the existing nanofluid model. The impacts of three different arrangements of conductive baffles on nanofluid motion and associated thermal behavior in a square geometry has been performed by Bendaraa et al.^35^ and noticed that the fin location has vital role in effective controlling of the flow movement and thus thermal dissipation rates. Buoyant nanofluid motion and the associated thermal dissipation rates are highly sensitive to shape of chosen geometry. In many applications, the geometrical configuration is not regularly-shaped and the convective transport rates can be effectively controlled through a vital geometrical parameter arising in non-regular geometries^36,37^. A detailed review and discussion on various constraints affecting nanofluid flow behavior and heat dissipation rate in different geometries and passages have been reported^38,39^. The above research works mainly focused on the convective flow of NFs but, the studies pertaining to the rheology of NFs are also very important in the application point of view. Experimental studies on rheological properties and the behavior of gold-NPs with poly (vinylidene fluoride) was examined in detail by Susrutha et al.^40^. Also, great amount of investigation on rheology and stability of NFs was addressed by Ram and co-workers^41,42^. The fascinating properties of magnetic NFs and its applications in magnetic resonance imaging, chromatography and many more has been illustrated in detail by Singh and Ram^43^.

In many practical situations, such as thermal bridge, heat barrier, design of thermal insulation, thermo couple design, gas turbine blade cooling, furnace design, design and sizing of heat exchangers, aerospace applications, the impact of wall thermal conduction should be taken into consideration, otherwise leads to inaccuracies in the prediction of flow movement and thermal behavior. Therefore, the impact of wall conduction on buoyant fluid motion and associated thermal removal from the hot boundary have received substantial attention by many theoretical and experimental analysis. In this direction, one of the pioneering and detailed study was made by Kaminski and Prakash^44^ in a square geometry by considering three different wall conduction models. For larger thermal gradients, they observed asymmetric flow field and non-uniformity in temperature along the interface between solid and fluid. Ben-Nakhi and Chamkha^45^ performed numerical analysis to understand the influence of a slim tilted baffle on conjugate buoyant convective motion in a square geometry having finite thickness on three boundaries. In a 2D square section, with thickness on its side wall, the change in flow motion and thermal behavior of liquid gallium subjected to an externally imposed tilted magnetic force and wall conductivity was examined by Belazizia et al.^46^ and reported that magnetic angle is a crucial parameter in controlling thermal transport. The impact of width of conductive walls and thermal conductivity ratio on natural convection in a porous square was analyzed by Saeid^47^. A detailed investigation on conjugate buoyant motion in finite porous geometry has been addressed by considering discrete heating effects^48^.

Conjugate buoyant motion and transport rate due to the presence of a solid block in an inclined^49^ and non-inclined^50,51^ square geometry was numerically analyzed in the presence and absence of magnetic field. By adopting Buongiorno’s model, Sheremet and Pop^52^ analyzed the Brownian movement and thermophoresis effect of NFs for a vast range of critical parameters and determined the range of parameters at which the chosen model could be used. Alsabery et al.^53^ discussed the impacts of non-uniform thermal conditions on nanofluid buoyant motion in a square geometry with thick bottom wall. The conjugate flow and thermal behavior of NFs undergo predominant changes due to the presence of protruding source or block in a finite geometry^54,55^. Ghalambaz et al.^56^ addressed the conjugate buoyant motion and thermal transport of hybrid NFs in a square geometry and discussed the impacts of all key parameters. Reddy and Narasimham^57^ performed numerical simulations to study combined conduction-convection in an enclosed annular region between inner heated rod and an outer cylinder for vast ranges of thermal conductivity ratios and presented thermal transfer correlations. Later, conjugate buoyant transport in a porous material placed in the annular region between two solid cylinders was numerically analyzed by Badruddin et al.^58^. Recently, John et al.^59^ made a detailed review on conjugate thermal transfer analysis covering various applied and theoretical aspects.

We made a meticulous and systematic literature survey on conjugate buoyant convection of base fluids as well as NFs in different shapes of finite geometries by considering most of the additional constraints. From the detailed survey of theoretical and experimental investigations, we have noticed that the conjugate buoyant motion and thermal transport behavior of NFs inside the enclosed annular region has not been analyzed so far. Therefore, keeping the applications involving thermal bridge or heat barrier in mind, a detailed numerical investigation is carried out to explore the impacts of various key parameters on flow and thermal behavior of three different NFs in the annular geometry having finite thickness at the inner cylinder.

## Formulation of problem and numerical procedure

### Formulation of problem

**Figure 1:**
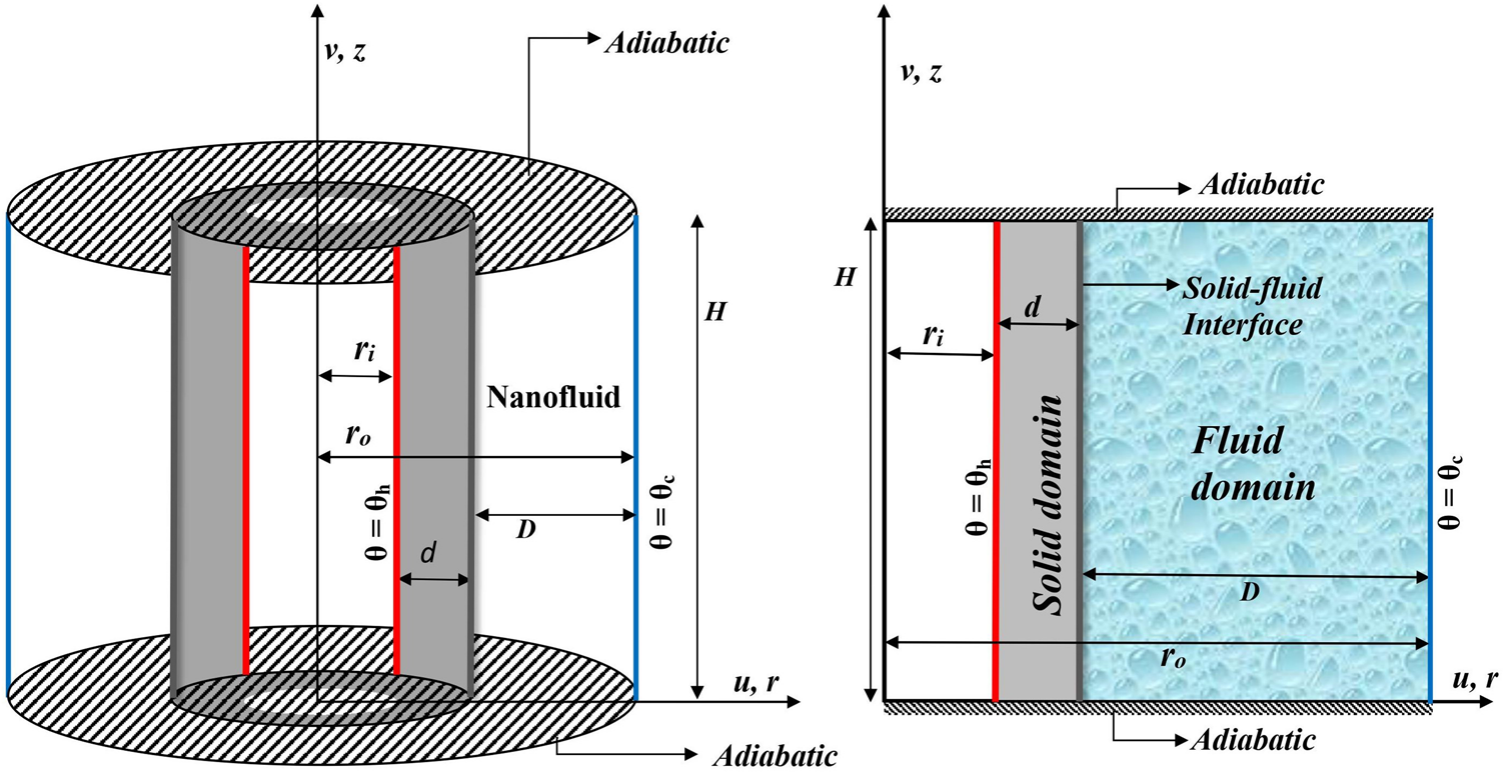
Physical model of conjugate natural convection in an annular geometry and the axi-symmetric view.

The geometrical structure, as portrayed in Fig. 1, is the enclosed concentric annulus region between two upright cylindrical tubes with solid inner cylinder of thickness ($\varepsilon$). The thermal conditions along the vertical boundaries are maintained such that the inner boundary is hotter than the outer surface, while at the horizontal surfaces, adiabatic condition is imposed. The annular gap is occupied by the water (H$_2$O)-based NFs having different types of NPs, such as Al$_2$O$_3$, TiO$_2$, Cu. Nanofluid is taken as a working medium in the annular region. It is assumed that the water and NPs are in thermal equilibrium. Thermo-physical properties of water and NPs are given in Table 1. Also, by neglecting the variation of fluid properties in angular or azimuthal direction, the flow analysis reduces to laminar, two-dimensional and axi-symmetric. The Boussinesq approximation which accounts the density variation in the body force term and constant elsewhere of momentum equation is utilized in this analysis. Further, the solid and fluid thermal conductivities are considered to be different. By utilizing these postulates, the governing equations are:

1 $$\begin{aligned} \nabla \cdot \vec{q} = 0, \end{aligned}$$

2 $$\begin{aligned} \rho _{nf}\left[ \displaystyle \frac{\partial \vec{q}}{\partial t^{*}}+(\vec{q} \cdot \nabla ) \vec{q}\right]= -\nabla p+\mu _{nf} \nabla ^{2}\vec{q}+(\rho \beta )_{nf}\vec{g} (\theta _{nf}-\theta _{c}),\end{aligned}$$

3 $$\begin{aligned} \displaystyle \frac{\partial \theta _{nf}}{\partial t^{*}}+(\vec{q} \cdot \nabla ) \theta _{nf}= \alpha _{nf} \nabla ^{2}\theta _{nf},\end{aligned}$$

4 $$\begin{aligned} \displaystyle \frac{\partial \theta _{w}}{\partial t^{*}}= \alpha _{w} \nabla ^{2}\theta _{w}. \end{aligned}$$

In the present study, thermophysical properties of NFs are described as below:^20^

$$\begin{aligned} \rho _{nf} & = (1-\phi )\rho _{f}+\phi \rho _{p},\\ (\rho \beta )_{nf} & = (1-\phi )(\rho \beta )_{f}+\phi (\rho \beta )_{p},\\ (\rho C_{p})_{nf} & = (1-\phi )(\rho C_{p})_{f}+\phi (\rho C_{p})_{p},\\ \frac{k_{nf}}{k_{f}} & = \frac{k_{p}+2k_{f}-2\phi (k_{f}-k_{p})}{k_{p}+2k_{f}+\phi (k_{f}-k_{p})},\\ \mu _{nf} & = \displaystyle \frac{\mu _{f}}{(1-\phi )^{2.5}},\\ \alpha _{nf} & = \frac{k_{nf}}{(\rho C_{p})_{nf}}. \end{aligned}$$

Here, the subscripts *nf*, *f* and *p* represents the nanofluid, fluid and nanoparticle, respectively. Applying the following transformations,

$$\begin{aligned} \displaystyle \left( R,Z\right) & =\frac{\left( r,z\right) }{D}, \quad \varepsilon =\displaystyle \frac{d}{D}, \quad U=\displaystyle {\frac{uD}{\alpha _{f}}}, \quad V=\displaystyle {\frac{vD}{\alpha _{f}}}, \quad t=\displaystyle {\frac{t^{*}}{\left( D^{2}/\alpha _{f}\right) }}, \\ T_w & =\displaystyle {\frac{(\theta _{w}-\theta _{c})}{\left( \theta _{h} -\theta _{c}\right) }}, \quad T_{nf} =\displaystyle {\frac{(\theta _{nf}-\theta _{c})}{\left( \theta _{h} -\theta _{c}\right) }}, \quad P = \displaystyle {\frac{p}{\rho _{nf}\left( \alpha _{f}/D\right) ^{2}}} , \end{aligned}$$

we finally get the dimensionless governing equations:

5 $$\begin{aligned} \frac{\partial U}{\partial R}+\frac{\partial V}{\partial Z} +\frac{U}{R}= 0, \end{aligned}$$

6 $$\begin{aligned} \frac{\partial U}{\partial t}+U\frac{\partial U}{\partial R}+V\frac{\partial U}{\partial Z}= -\frac{\partial P}{\partial R}+\frac{\mu _{nf}}{\rho _{nf}\alpha _{f}}\left[ \frac{\partial ^{2} U}{\partial R^{2}}+\frac{1}{R}\frac{\partial U}{\partial R}+\frac{\partial ^{2} U}{\partial Z^{2}}-\frac{U}{R^{2}}\right] ,~\end{aligned}$$

7 $$\begin{aligned} \frac{\partial V}{\partial t}+U\frac{\partial V}{\partial R}+V\frac{\partial V}{\partial Z}= -\frac{\partial P}{\partial Z}+\frac{\mu _{nf}}{\rho _{nf}\alpha _{f}} \left[ \frac{\partial ^{2} V}{\partial R^{2}}+\frac{1}{R}\frac{\partial V}{\partial R}+ \frac{\partial ^{2} V}{\partial Z^{2}}\right] +\frac{(\rho \beta )_{nf}}{\rho _{nf}\beta _{f}}~Ra~Pr~T_{nf},~\end{aligned}$$

8 $$\begin{aligned} \frac{\partial T_{nf}}{\partial t}+U\frac{\partial T_{nf}}{\partial R}+V\frac{\partial T_{nf}}{\partial Z}= \frac{\alpha _{nf}}{\alpha _{f}}\left[ \frac{\partial ^{2} T_{nf} }{\partial R^{2}}+\frac{1}{R}\frac{\partial T_{nf}}{\partial R}+ \frac{\partial ^{2} T_{nf}}{\partial Z^{2}}\right] ,\end{aligned}$$

9 $$\begin{aligned} \frac{\partial T_{w}}{\partial t}= \frac{\alpha _{w}}{\alpha _{f}}\left[ \frac{\partial ^{2} T_{w} }{\partial R^{2}}+ \frac{1}{R}\frac{\partial T_{w}}{\partial R}+\frac{\partial ^{2} T_{w}}{\partial Z^{2}}\right] , \end{aligned}$$

where $\displaystyle {Ra=\frac{g\beta _{f}\Delta \theta D^{3}}{\nu _{f}\alpha _{f}}}$ and $\displaystyle {Pr=\frac{\nu _{f}}{\alpha _{f}}}$ are the Rayleigh and Prandtl numbers, respectively.

**Table 1:** Thermo-physical properties of water and nanoparticles.

Property	H$_2$O	Cu	Al$_2$O$_3$	TiO$_2$
$\rho$ (kg/$\hbox {m}^3$)	997.1	8933	3970	4250
$C_{p}$ (J/kg K)	4179	385	765	686.2
*k* (W/mK)	0.613	401	40	8.9538
$\beta$ ($\hbox {K}^{-1}$)	$21\times 10^{-5}$	$1.67 \times 10^{-5}$	$0.85 \times 10^{-5}$	$0.9 \times 10^{-5}$

The numerical values are taken from Roy^33^.

By introducing two-dimensional stream function $\psi (R,Z)$, the momentum equations (6) and (7) can be expressed in the following vorticity-stream function form:

10 $$\begin{aligned} \frac{\partial \zeta }{\partial t}+U \frac{\partial \zeta }{\partial R}+ V \frac{\partial \zeta }{\partial Z}-\frac{U\zeta }{R}= & {} \frac{\mu _{nf}}{\rho _{nf}\alpha _{f}}\left[ \nabla ^2\zeta -\frac{\zeta }{R^2}\right] -\frac{\left( \rho \beta \right) _{nf}}{\rho _{nf}\beta _{f}}~Ra ~Pr ~\frac{\partial T_{nf}}{\partial R}, \end{aligned}$$

11 $$\begin{aligned} \zeta= \frac{1}{R}\left[ \frac{\partial ^2 \psi }{\partial R^2} -\frac{1}{R}\frac{\partial \psi }{\partial R}+\frac{\partial ^2 \psi }{\partial Z^2} \right] ,\end{aligned}$$

12 $$\begin{aligned} U= \frac{1}{R}\frac{\partial \psi }{\partial Z},\quad V = -\frac{1}{R}\frac{\partial \psi }{\partial R}, \end{aligned}$$

where $$\displaystyle \nabla ^2 = \frac{\partial ^2 }{\partial R^2}+\frac{1}{R}\frac{\partial }{\partial R}+\frac{\partial ^2 }{\partial Z^2}$$.

The initial and boundary conditions in dimensionless form are;

$$\begin{aligned} t=0{:}~~U= & {} V=T_w=T_{nf}=0,~\psi =\zeta =0; \quad ~\text{ at } \quad ~ 0\le Z \le A \quad ~\text{ and } \quad ~\frac{1}{\lambda -1} \le R \le \frac{\lambda }{\lambda -1}.\\ t>0{:}~~ \psi= & {} \frac{\partial \psi }{\partial R}=0,~T_{w}=1 \quad ~~\text{ at } \quad ~~R=\frac{1}{\lambda -1} \quad ~~\text{ and } \quad ~ 0\le Z \le A \\ \psi= & {} \frac{\partial \psi }{\partial R}=0,~T_{nf}=0 \quad \text{ at } \quad ~~R=\frac{\lambda }{\lambda -1} \quad ~~\text{ and } \quad ~ 0\le Z \le A\\ \psi= & {} \frac{\partial \psi }{\partial Z}=0,~ \frac{\partial T_{w}}{\partial Z}=~\frac{\partial T_{nf}}{\partial Z} = 0 \quad ~\text{ at }~ \quad Z=0 \quad ~\text{ and } \quad ~Z=A\\ \frac{\partial T_{nf}}{\partial R}= & {} Kr~\frac{\partial T_{w}}{\partial R}~ ~\text{ at } \text{ the } \text{ interface, } \end{aligned}$$

where $Kr=\displaystyle \frac{k_w}{k_{nf}}$ is the thermal conductivity ratio.

The thermal dissipation rates are measured through the average Nusselt numbers along the solid wall $({\overline{Nu}}_w)$ as well as at the interface $({\overline{Nu}}_i)$, and are given by

$$\begin{aligned} \displaystyle {{\overline{Nu}}_w=\frac{1}{A}\int \limits _{0}^{A}Nu_{w}~dZ} \ \ \hbox { and } \ \ \displaystyle {{\overline{Nu}}_i=\frac{1}{A}\int \limits _{0}^{A}Nu_{i}~dZ}, \end{aligned}$$

where $$\begin{aligned} Nu_{w}=-\displaystyle \left( \frac{\partial T}{\partial R}\right) _{R=\frac{1}{\lambda -1}} \end{aligned}$$ and    $$\begin{aligned} Nu_{i}=-\displaystyle \frac{k_{nf}}{k_{f}}\left( \frac{\partial T}{\partial R}\right) _{R=\varepsilon }. \end{aligned}$$

### Numerical methodology

The coupled and nonlinear partial differential equations (PDEs) and associated supplementary conditions governing the physical processes are numerically solved by utilizing a suitable implicit finite difference method (FDM). In particular, the transient PDEs, such as vorticity and temperature equations are discretized using alternating direction implicit (ADI) method and successive line over relaxation (SLOR) method is adopted to solve steady-state stream function equation^21,22,60^. These FDM based techniques reduce the PDEs to a system of linear algebraic FD equations with tridiagonal structure and using tri-diagonal matrix algorithm (TDMA), the solutions are obtained. We have performed the grid independence trials by considering five different grid sizes, varying from sparse to finer grids, as shown in Table 2. ${\overline{Nu}}_w$ and ${\overline{Nu}}_i$ are measured for each grid size and used as the sensitivity measure to check the optimum grid size. We did not detect a considerable change in ${\overline{Nu}}_w$ and ${\overline{Nu}}_i$ between $201\times 201$ and $251\times 251$ grid sizes. Therefore, by accounting accuracy and computation time, a grid size of $201\times 201$ is utilized for all simulations of present investigation. An in-house FORTRAN code was written and few verifications are executed to validate the simulation results.

**Table 2:** Grid independence test for $Ra=10^6$, $\varepsilon =0.4$, $Kr=10$ and $\phi =0.2$.

Grid size	$\overline{Nu_{w}}$	$\overline{Nu_{i}}$
$81 \times 81$	0.9875297	9.0849894
$101 \times 101$	0.9902507	9.0542851
$161 \times 161$	0.9939314	9.0221870
$201 \times 201$	0.9950641	9.0145831
$251 \times 251$	0.9952458	9.0139465

**Table 3:** Comparison of the average Nusselt number obtained from the present numerical simulations with the correlation results of Abouali and Falahatpishesh^18^ for different values of Rayleigh number.

*Ra* ($Gr\times Pr$)	Abouali and Falahatpishesh^18^	Present results	Difference (%)	
$6 \times 10^3$	2.8673	2.8843	0.59	$\phi =0.00$
	2.7291	2.7401	0.40	$\phi =0.02$
$6 \times 10^4$	5.4385	5.4796	0.75	$\phi =0.00$
	5.1762	5.1836	0.14	$\phi =0.02$
$6 \times 10^5$	10.3150	10.4015	0.83	$\phi =0.00$
	9.8178	9.9132	0.96	$\phi =0.02$

**Figure 2:**
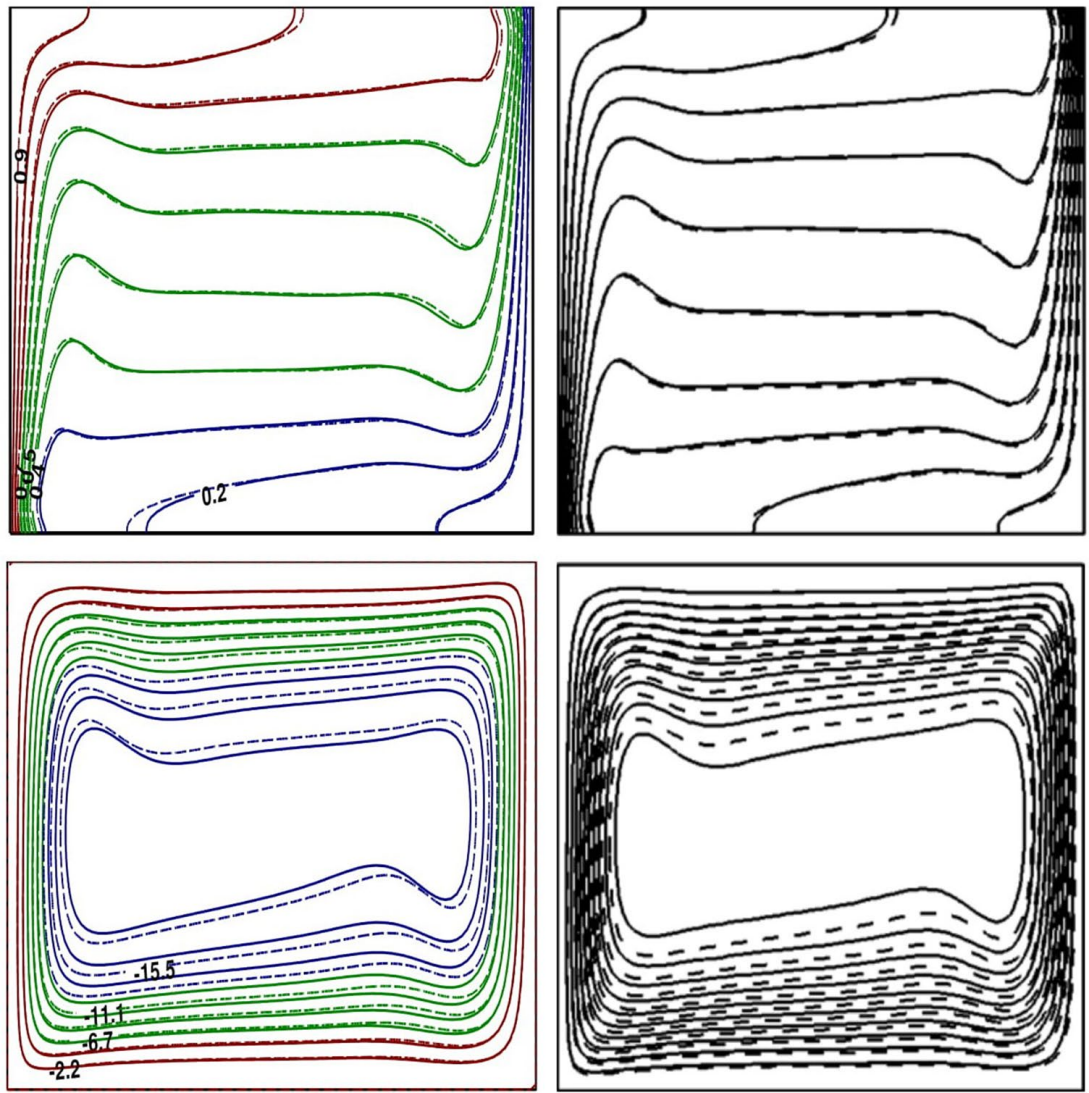
Comparison of simulations (left) with the results of Ho et al.^28^ (right). Base fluid (dotted line) and NF (solid line).

### Validation

The current simulations are tested with different benchmark data present in the literature. Firstly, the global heat transfer rate for an annular geometry containing Al$_{2}$O$_3$ based NF in the limiting case of zero thickness of inner cylinder is compared with present predictions. The relative difference in ${\overline{Nu}}$ between our predictions and those estimated from the correlation suggested by Abouali and Falahatpishesh^18^, displayed in Table 3, is in acceptable range for all *Ra* values. An additional quantitative comparison of flow and thermal contours in a square geometry containing Al$_{2}$O$_3$ based NF with $\phi =0.04$ at $Ra=10^{6}$, is generated by setting $\lambda = 1$ in the present analysis. An excellent qualitative comparison of isotherms and streamlines between the present results and Ho et al.^28^ analysis is exhibited in Fig. 2 and the agreement between two predictions is quite good. The excellent concurrence in the qualitative and quantitative comparison of our results with benchmark predictions for square and annular geometries ensure the credibility of developed code.

## Results and discussion

**Figure 3:**
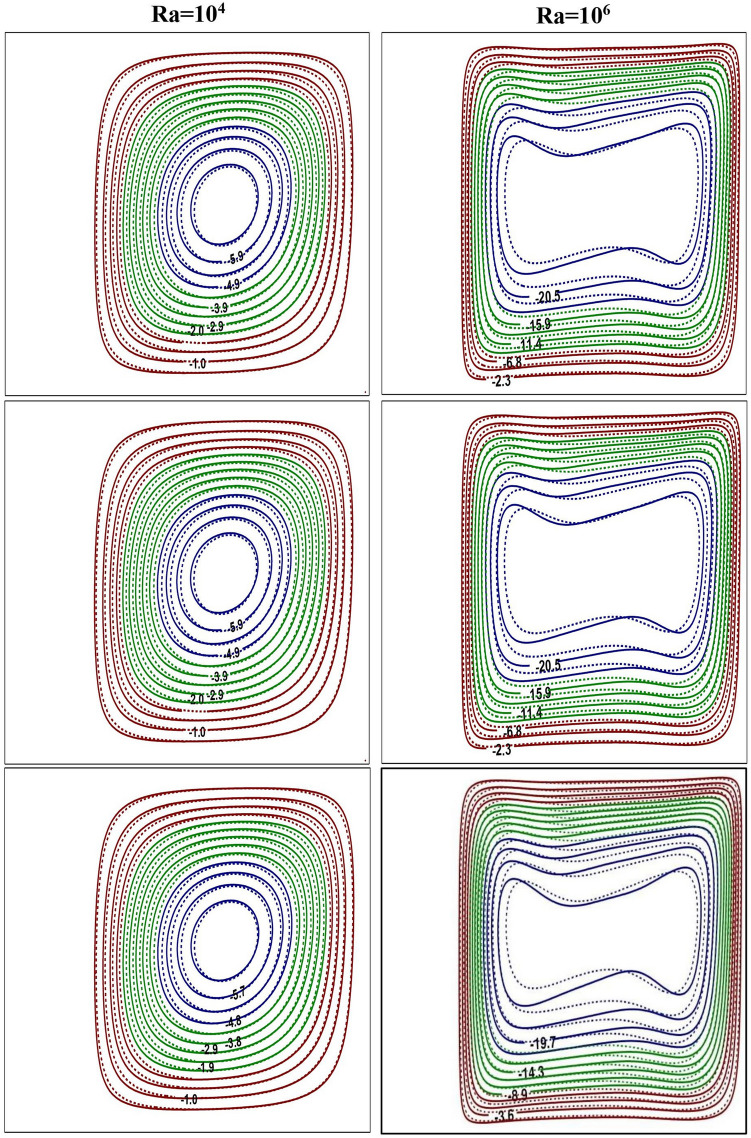
Impact of *Ra* on flow contours at $Ra=10^{4}$ (left) and $Ra=10^{6}$ (right) for base fluid (solid curve) with Al$_{2}$O$_{3}$-NF (top), TiO$_{2}$-NF (middle) and Cu-NF (bottom) (dotted curve), with $\phi =0.1$, $Kr=5$ and $\varepsilon =0.2$.

**Figure 4:**
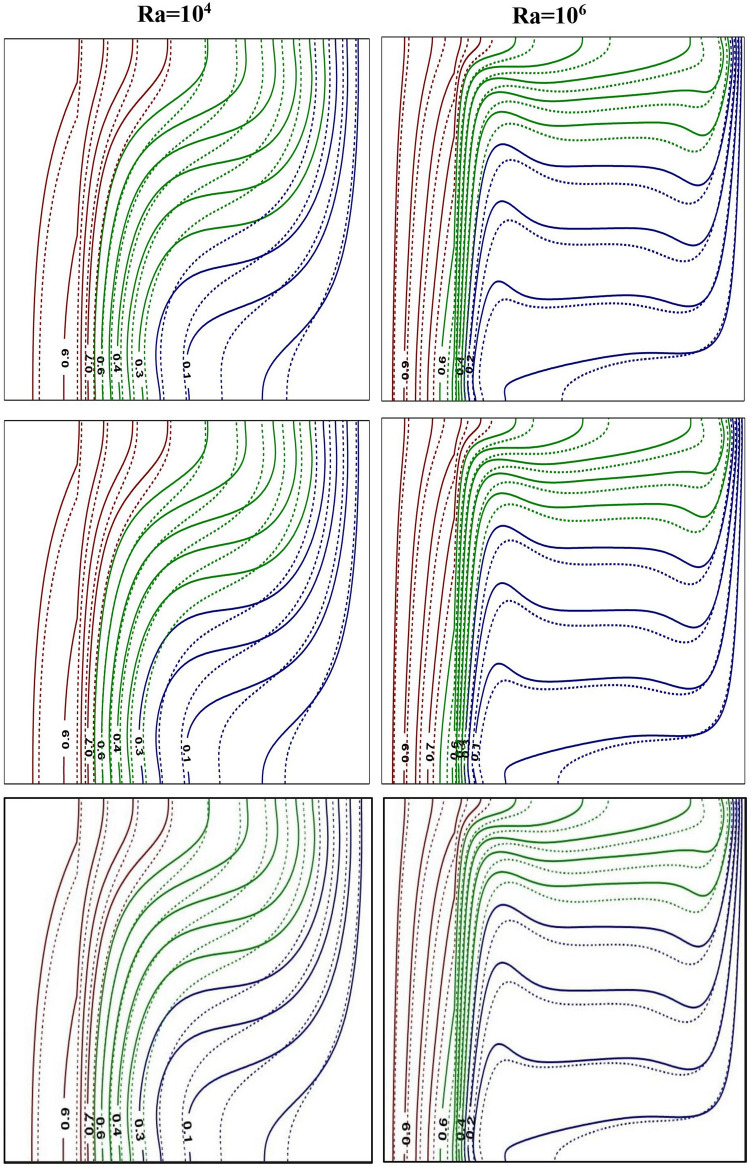
Impact of *Ra* on thermal contours at $Ra=10^{4}$ (left) and $Ra=10^{6}$ (right) for base fluid (solid curve) with Al$_{2}$O$_{3}$-NF (top), TiO$_{2}$-NF (middle) and Cu-NF (bottom) (dotted curve), with $\phi =0.1$, $Kr=5$ and $\varepsilon =0.2$.

**Figure 5:**
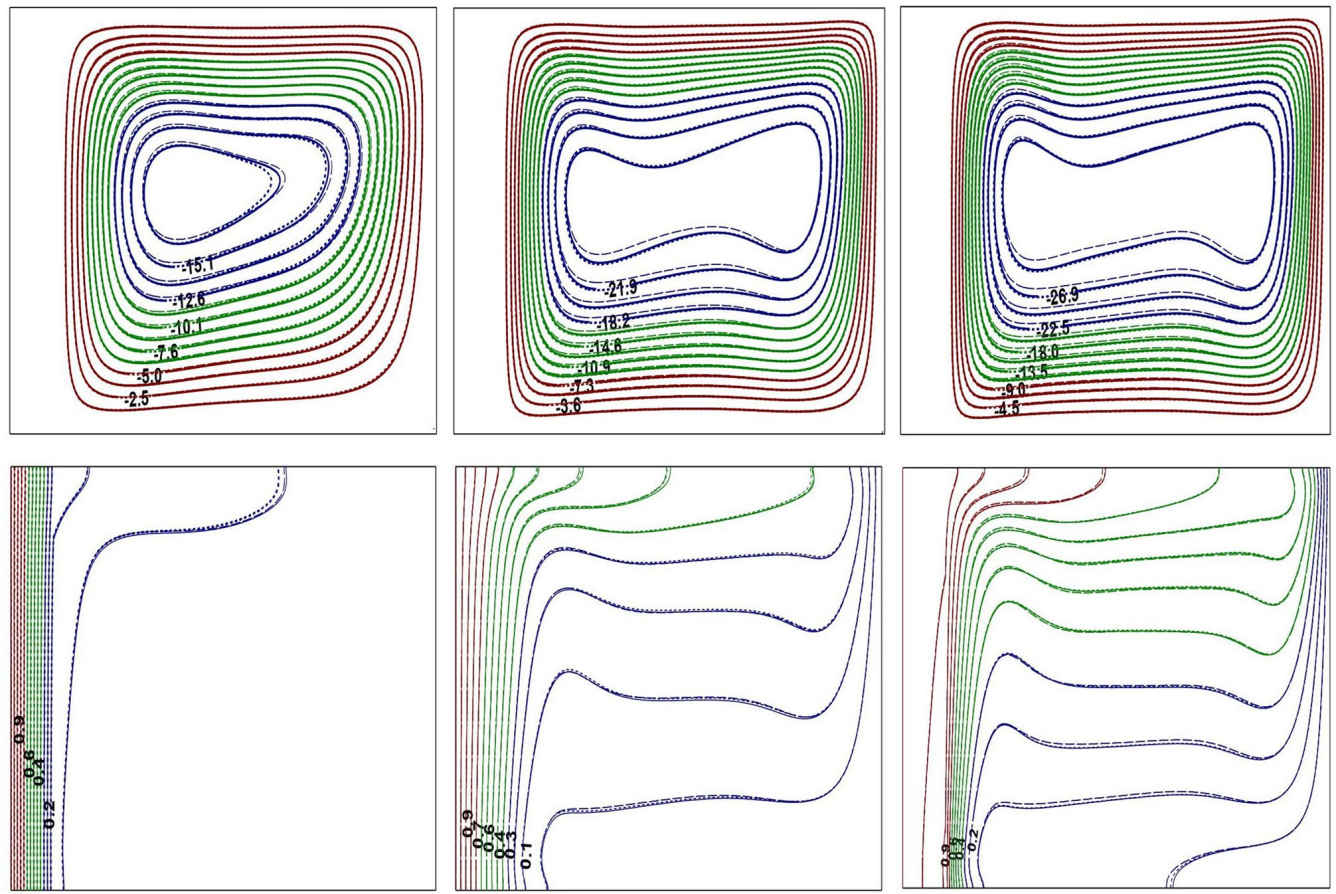
Impact of *Kr* on flow and thermal contours at $Ra=10^{6}$, $\phi =0.1$ and $\varepsilon =0.1$. $Kr=0.1$ (left), $Kr=1.0$ (center) and $Kr=10$ (right).

**Figure 6:**
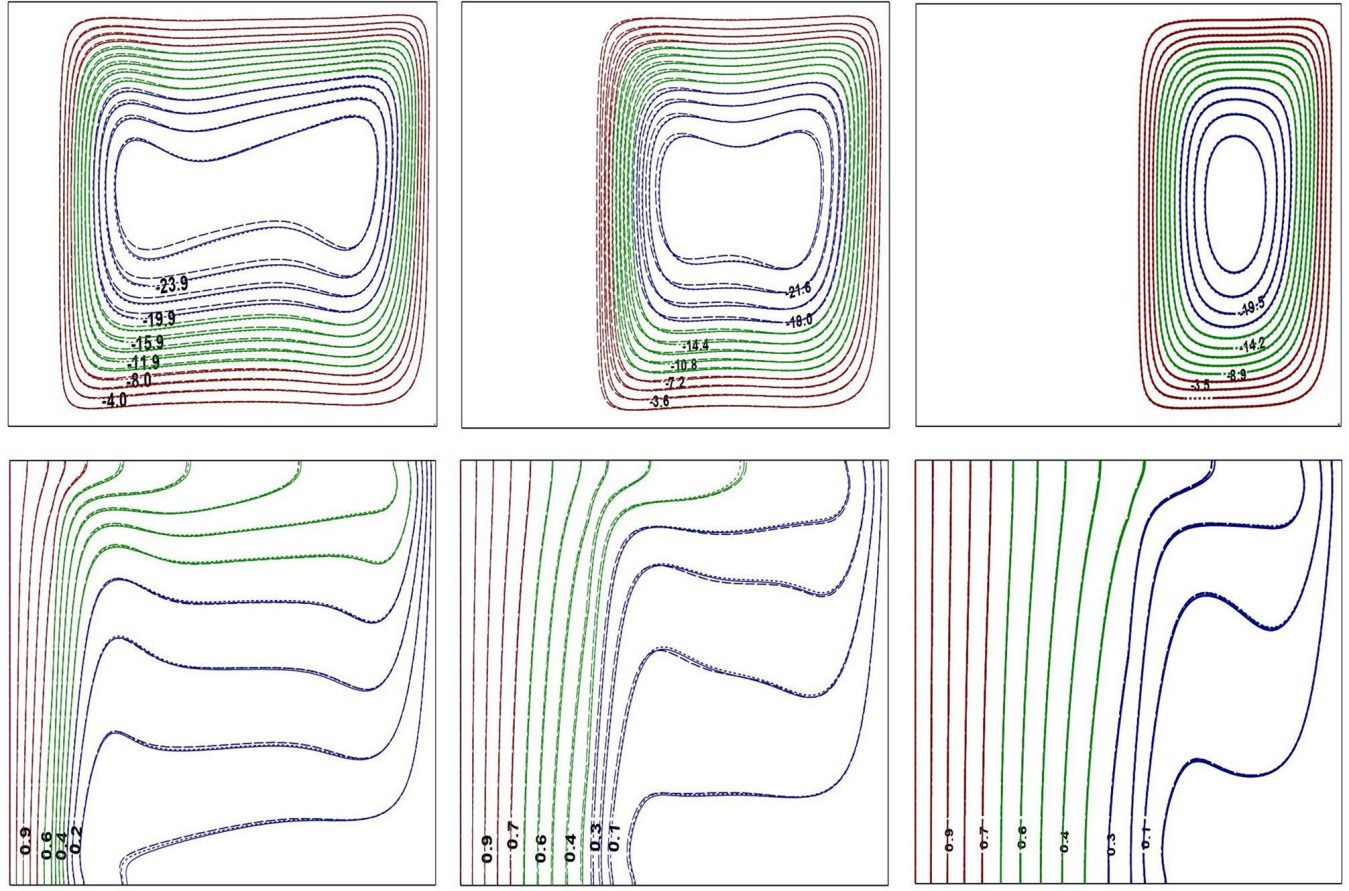
Impact of $\varepsilon$ on flow and thermal contours at $Ra=10^{6}$, $\phi =0.1$ and $Kr=2$. $\varepsilon =0.1$ (left), $\varepsilon =0.3$ (center) and $\varepsilon =0.5$ (right).

**Figure 7:**
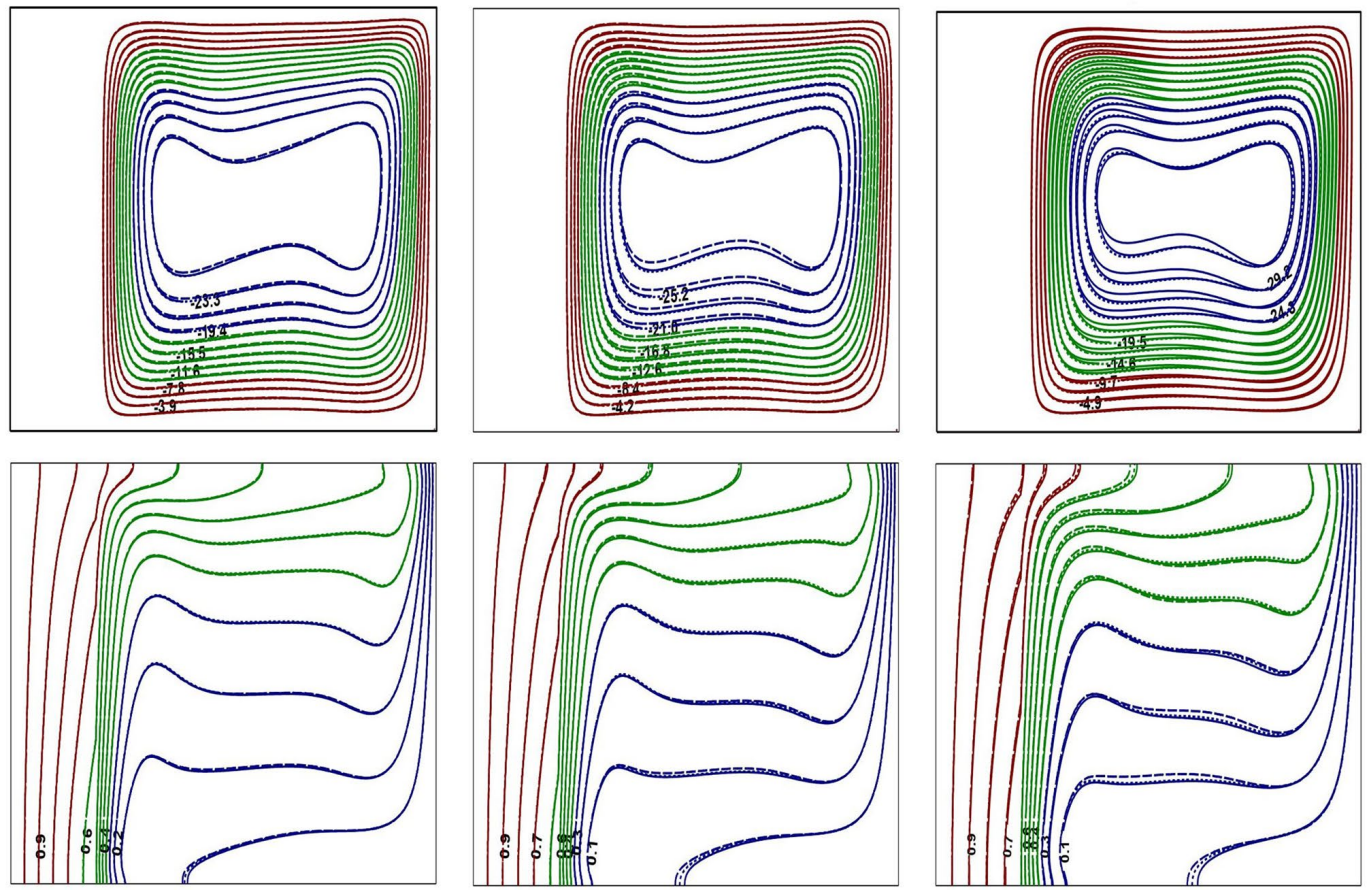
Impact of $\phi$ on flow and thermal contours at $Ra=10^{6}$, $\varepsilon =0.2$ and $Kr=5$. $\phi =0.05$ (left), $\phi =0.1$ (center) and $\phi =0.2$ (right).

**Figure 8:**
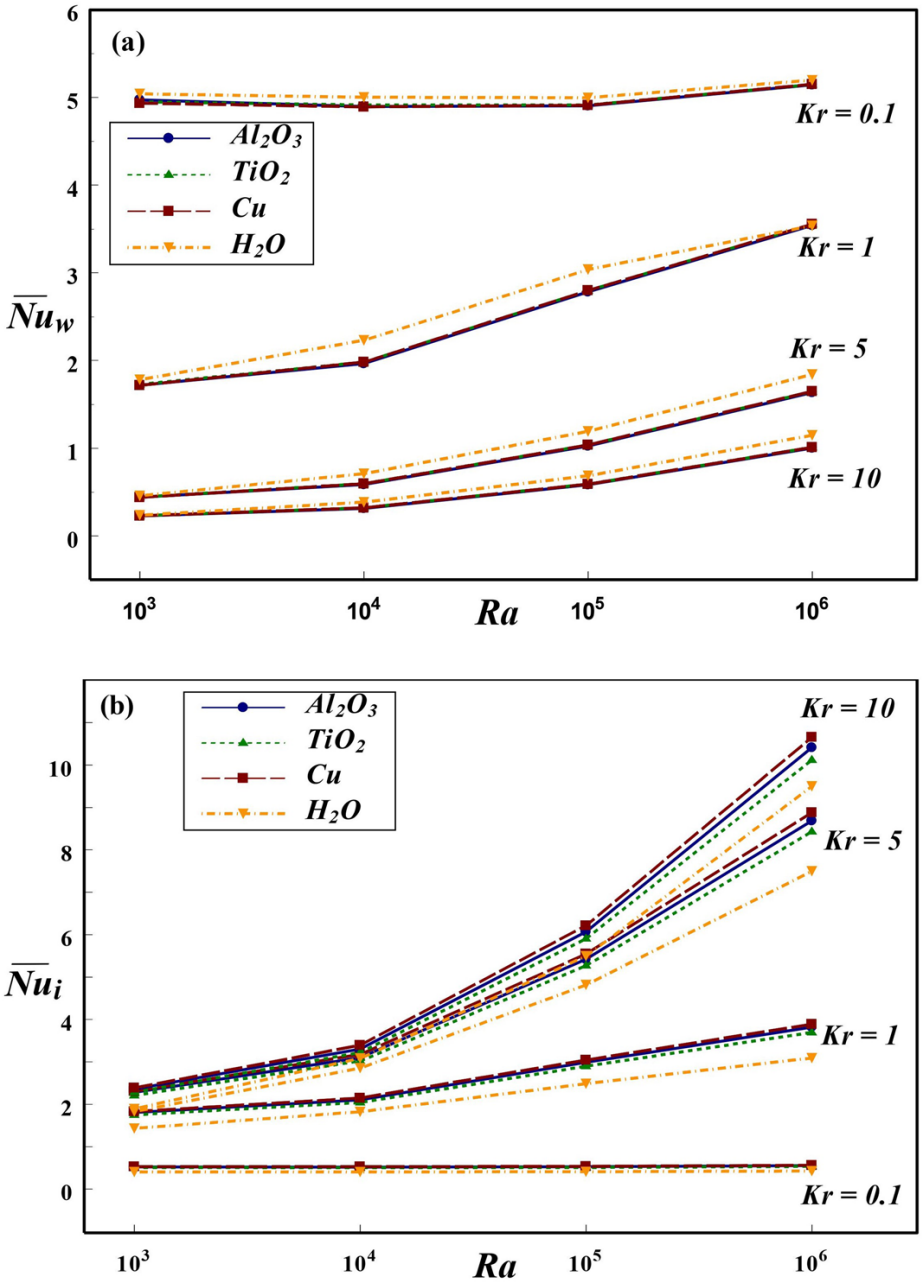
Effect of *Ra* and *Kr* on ${\overline{Nu}}$ at the (**a**) wall and (**b**) interface for $\varepsilon =0.2$ and $\phi =0.1$.

**Figure 9:**
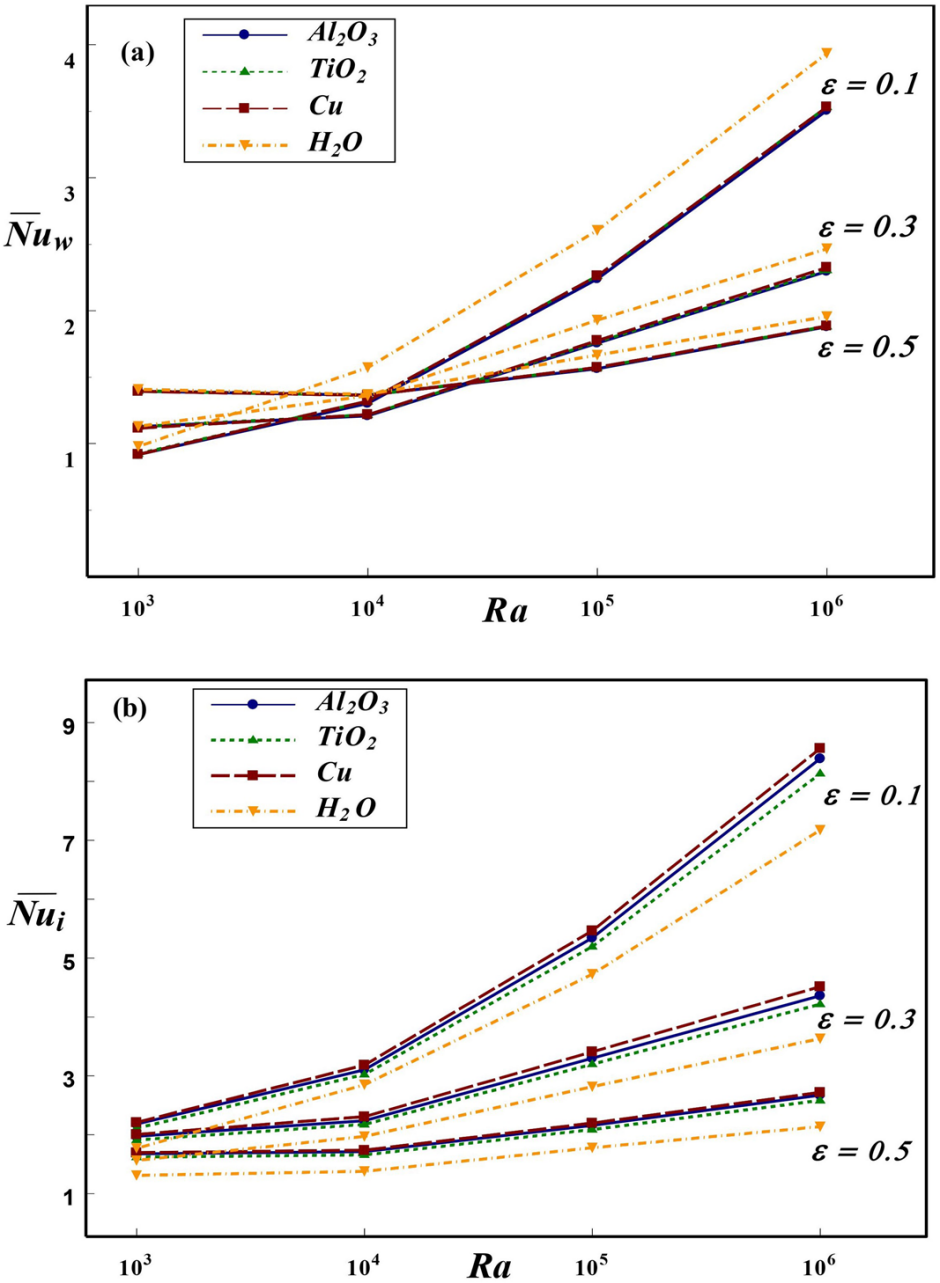
Effect of *Ra* and $\varepsilon$ on ${\overline{Nu}}$ at the (**a**) wall and (**b**) interface for $Kr=2$ and $\phi =0.1$.

**Figure 10:**
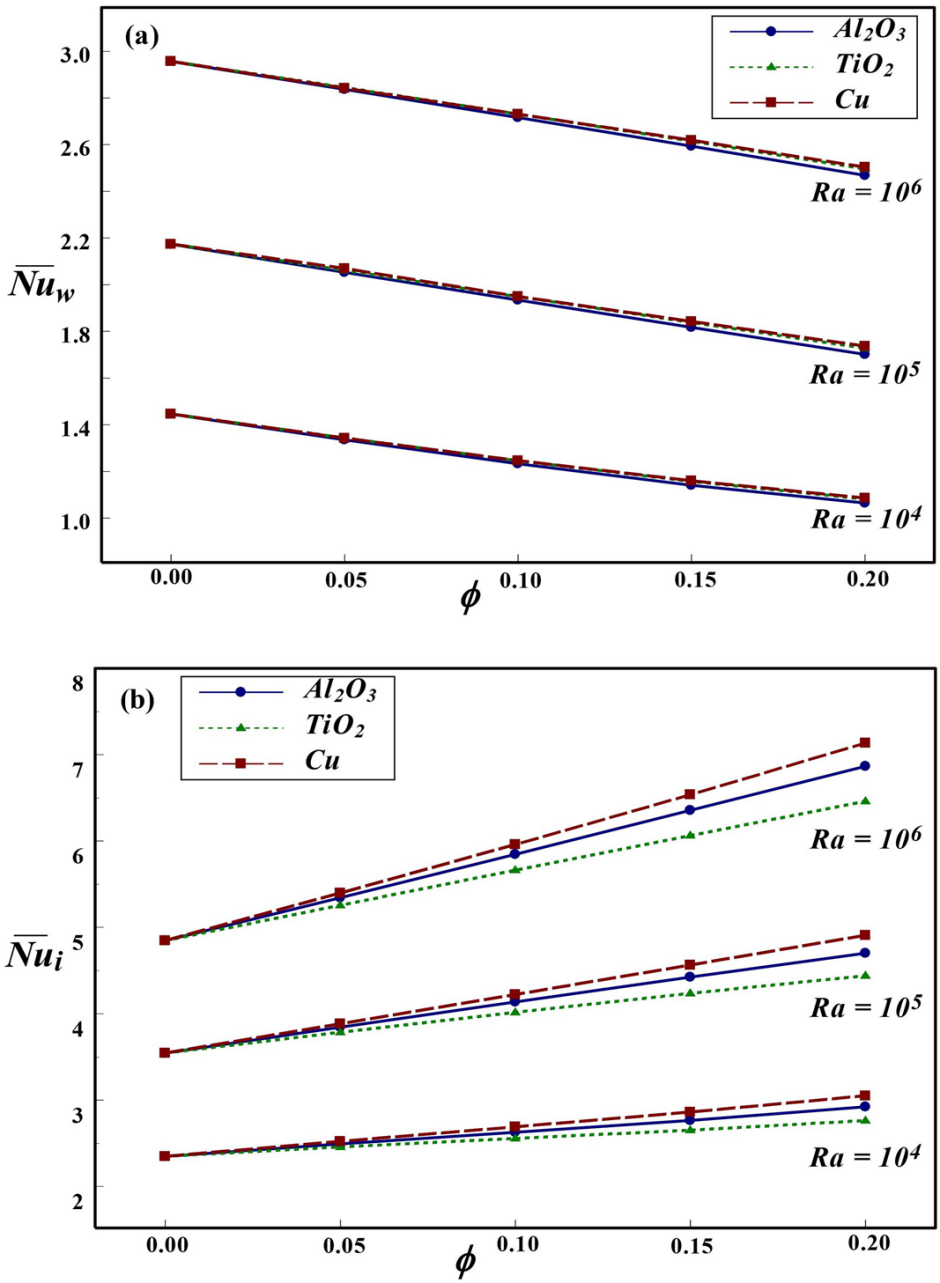
Effect of *Ra* and $\phi$ on ${\overline{Nu}}$ at the (**a**) wall and (**b**) interface for $Kr=2$ and $\varepsilon =0.2$.

**Figure 11:**
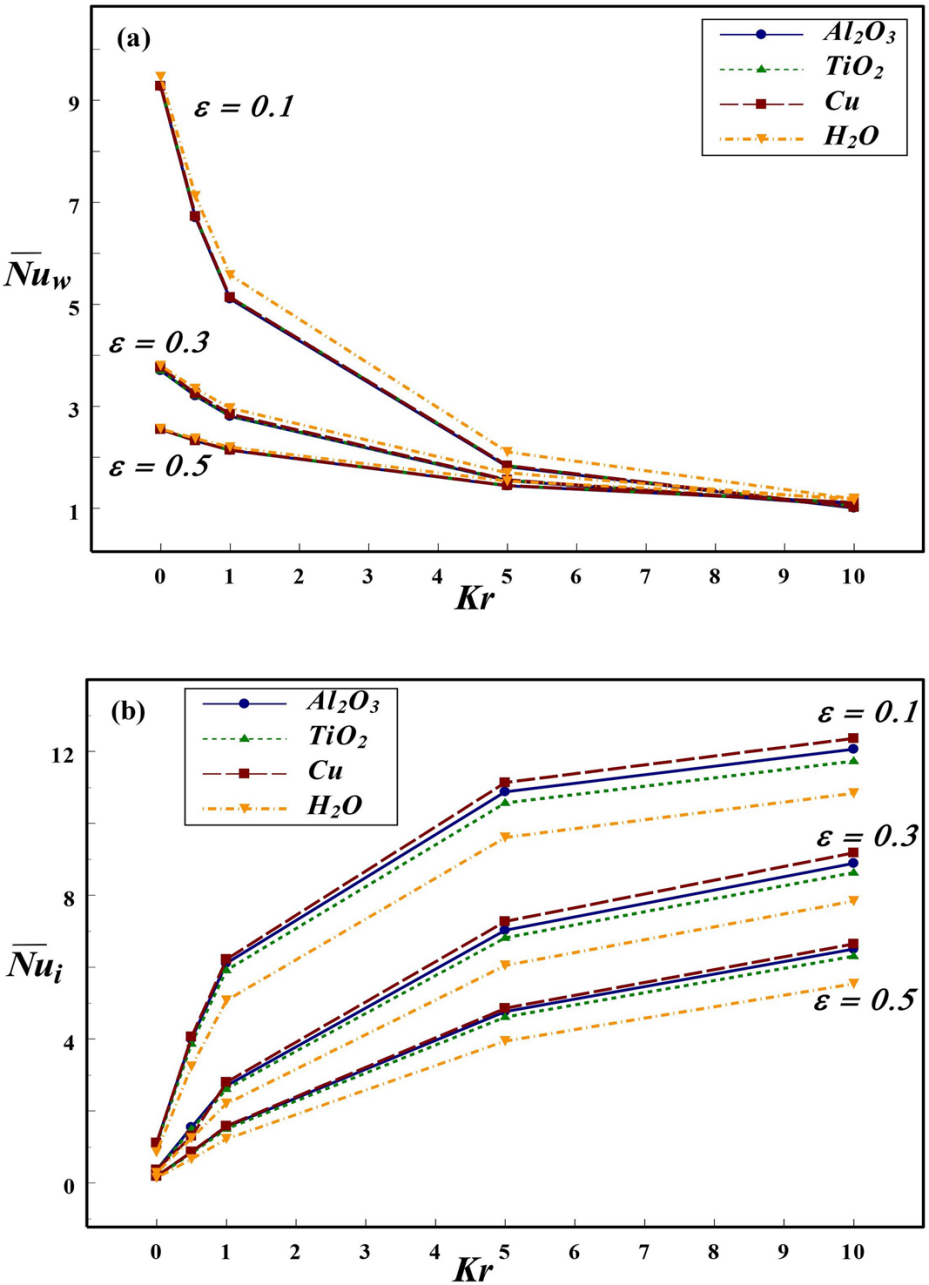
Effect of *Kr* and $\varepsilon$ on ${\overline{Nu}}$ at the (**a**) wall and (**b**) interface for $Ra=10^{6}$ and $\phi =0.1$.

**Figure 12:**
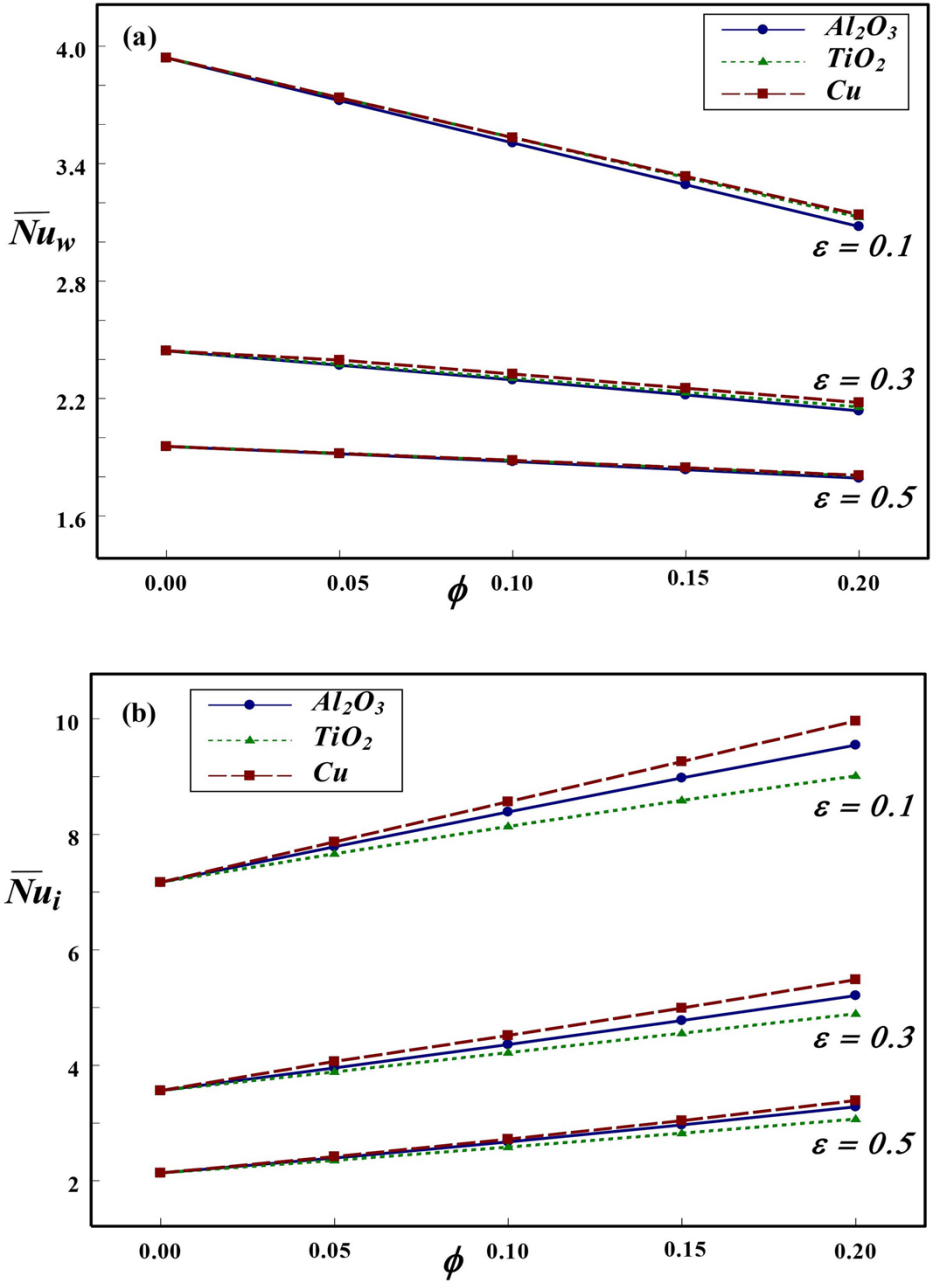
Effect of $\phi$ and $\varepsilon$ on ${\overline{Nu}}$ at the (**a**) wall and (**b**) interface for $Ra=10^{6}$ and $Kr=2$.

**Figure 13:**
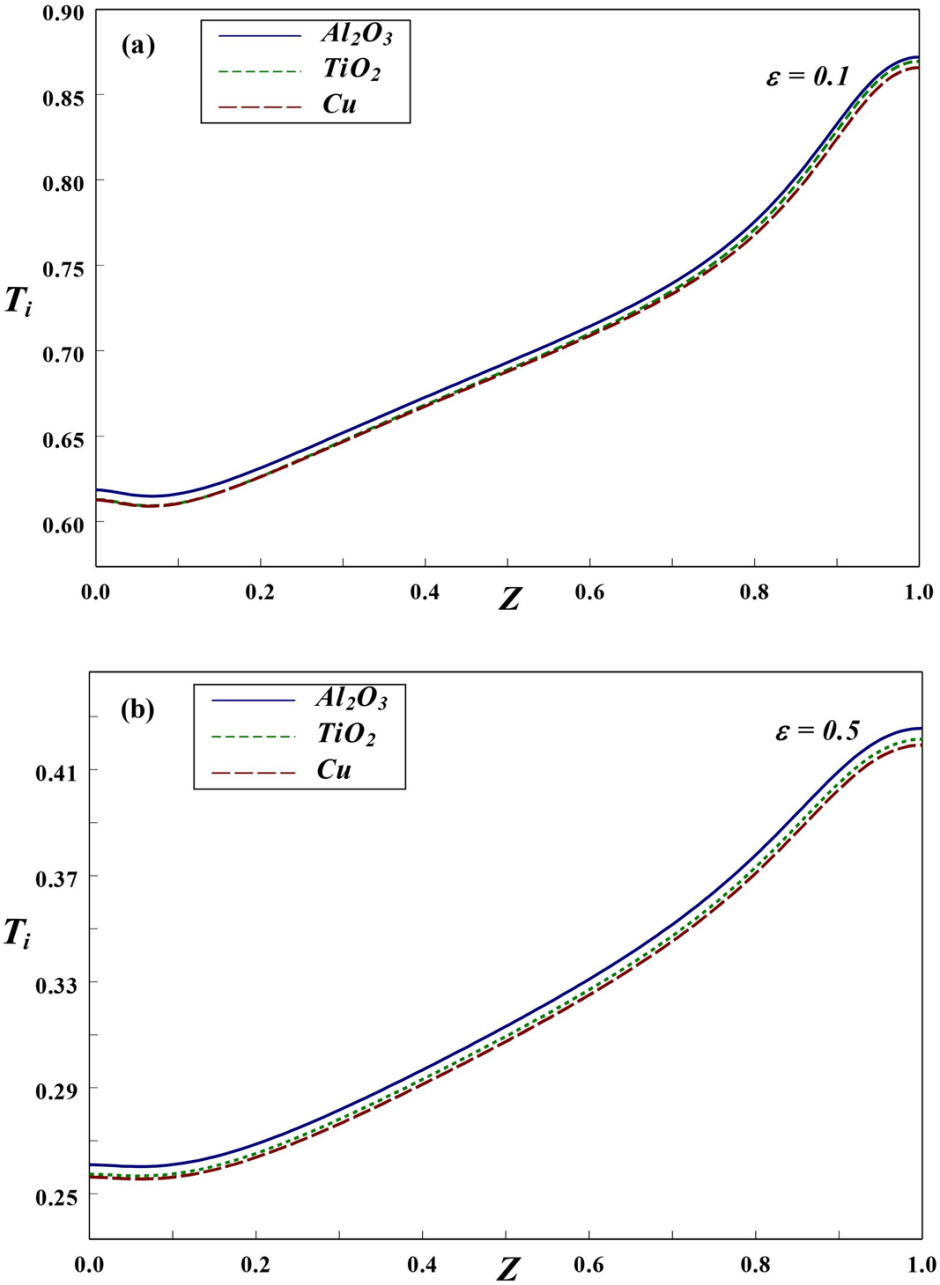
Axial variation of interface temperature with (**a**) $\varepsilon =0.1$ and (**b**) $\varepsilon =0.5$.

**Figure 14:**
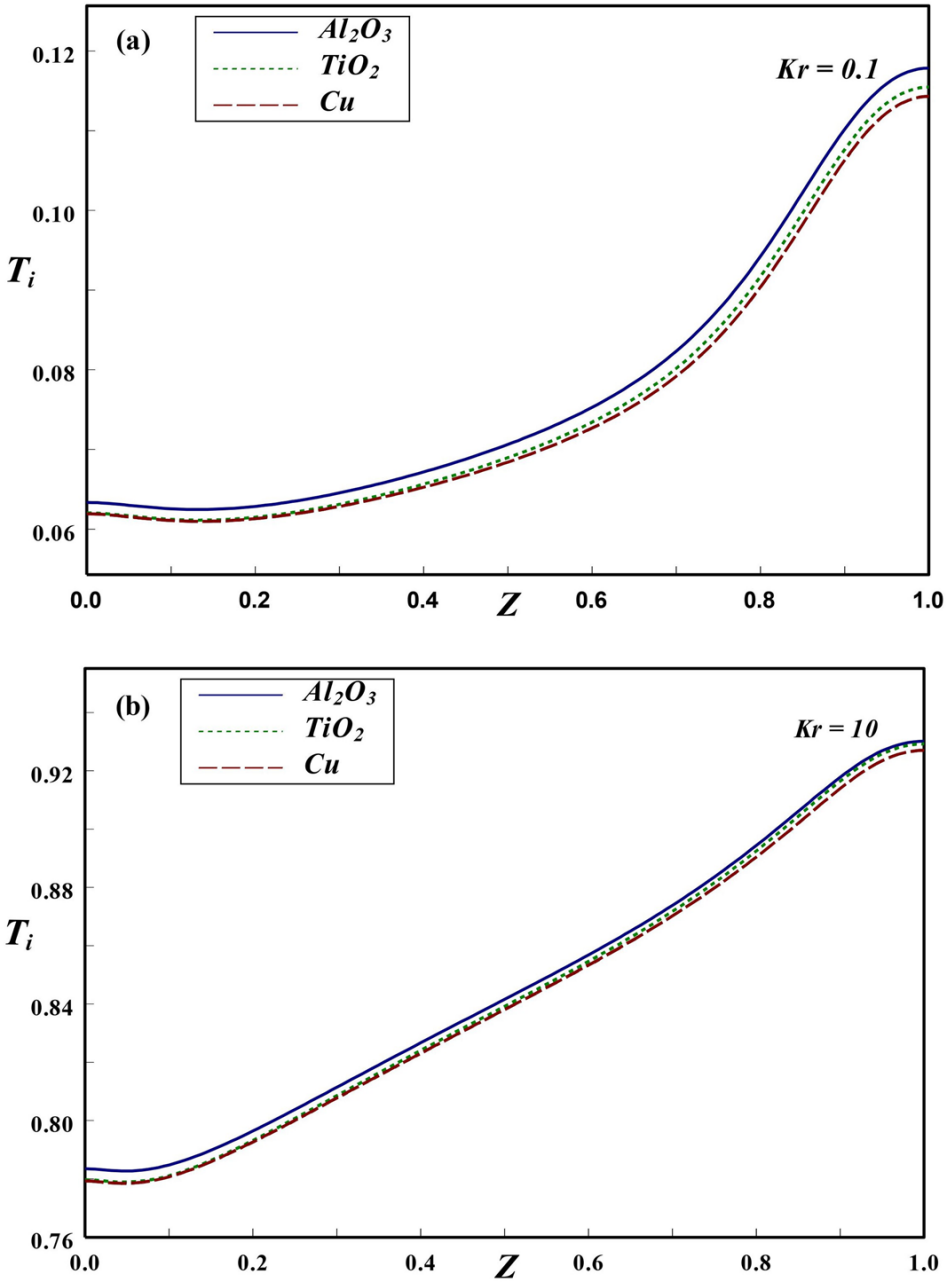
Axial variation of interface temperature with (**a**) $Kr=0.1$ and (**b**) $Kr=10$.

**Figure 15:**
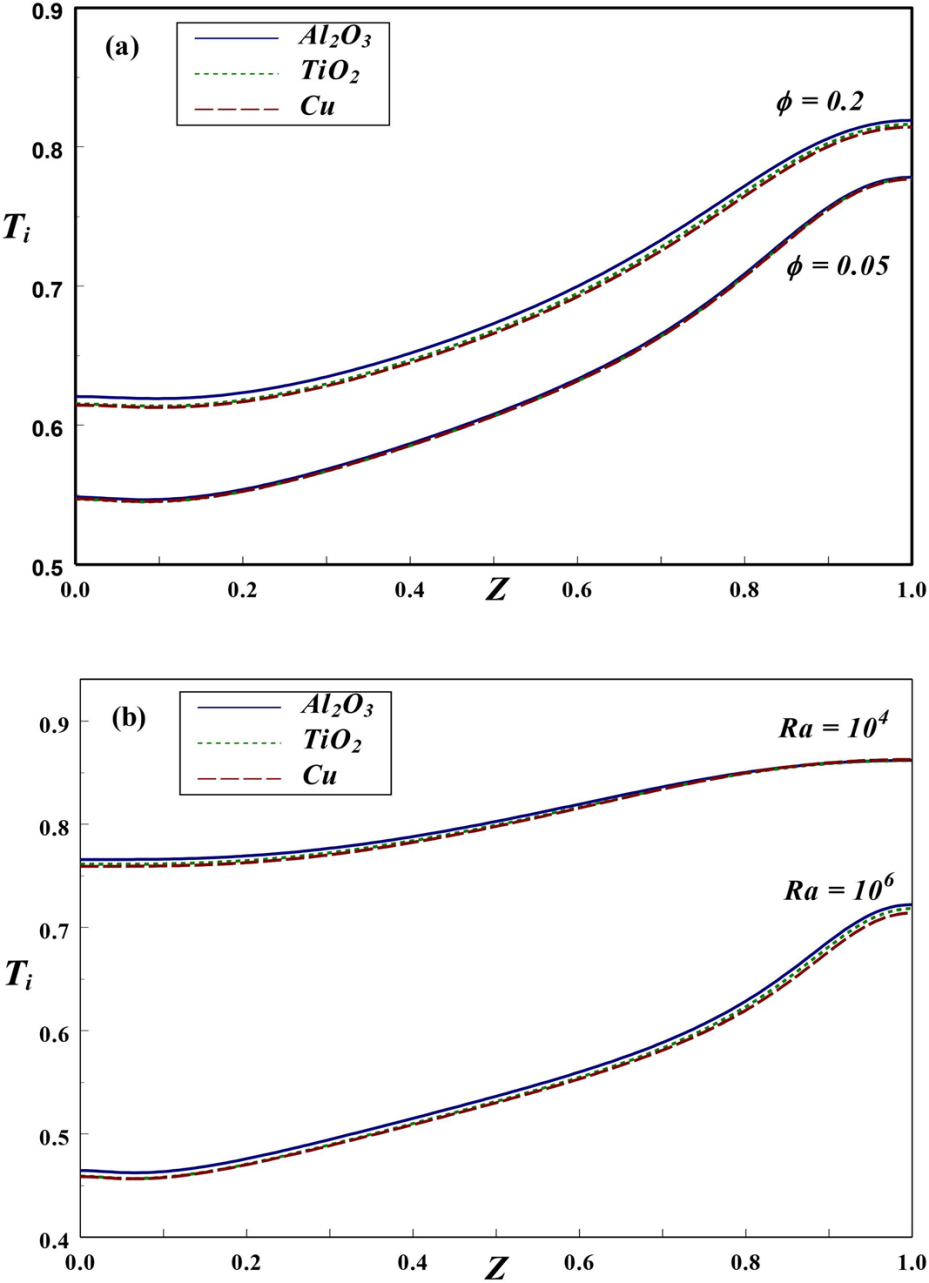
Axial variation of interface temperature with different (**a**) $\phi$ and (**b**) *Ra* values.

In the present analysis, the combined conduction-convective flow and associated thermal transport analysis is performed in the solid and annular regions. The dimensionless parameters arising in this investigation, varied over wide spectrum of values, $0.1 \le \varepsilon \le 0.5$, $0.1 \le Kr \le 10$, $10^3 \le Ra \le 10^6$ and $0 \le \phi \le 0.2$. For all simulations, the values of Prandtl number and radius ratio are fixed at $Pr=6.2$ and $\lambda =2$ respectively. The impact of these parameters on buoyant motion of three different NFs in the annular domain has been investigated. The simulations are displayed through the flow and thermal contours, temperature profiles, and global thermal dissipation rates.

Figures 3 and 4 represent the impacts of *Ra* on flow and thermal contours for three different NFs and base-fluid by keeping $Kr=5$ and $\varepsilon =0.2$. For lower $Ra=10^4$, the conventional single-eddy flow structure with moderate circulation rate is noticed and slightly distorted isothermal contours with less stratification indicates conduction-dominated heat transfer with minimal convective transport. With increasing *Ra* to a maximum chosen value $10^6$, a threefold enhancement in the strength of nanofluid buoyant motion is observed. The streamline vortex pattern has been modified with an enhanced $Ra=10^6$. Flow and thermal contour pattern for all three NFs, considered in this simulations, have not changed appreciably. However, though the streamlines appear to be similar for all three NFs, a minor change in fluid flow strength is detected. At lower *Ra* $(Ra=10^4)$, the isotherms are almost parallel to the vertical walls which implies the dominance of conduction heat transfer mechanism in the annular enclosure, whereas at higher *Ra* $(Ra=10^6)$, the stratification of isotherms can be observed. At this *Ra*, the temperature difference between the vertical walls increases which leads to more convection in the fluid region. Thus, the isotherms become well distorted at higher *Ra* and this indicates that the conduction heat transfer at lower *Ra* has been changed to convective heat transfer for $Ra=10^6$. At higher *Ra*, the isotherms are horizontally skewed in the fluid region due to convection-dominant state and larger thermal gradients are noticed near the solid–fluid interface. Similar isothermal structure pattern is detected for all the samples.

In Figs. 5, 6, and 7, solid, dotted and dashed curves represent Al$_2$O$_3$, TiO$_2$ and Cu-NFs, respectively. Since the analysis involves fluid region along with solid portion, the impact of thermal conductivities of both solid and nanofluid need to be considered. The impact of *Kr* on streamlines and isotherms is depicted in Fig. 5 for all three NFs. For poor thermal conductivity of the wall ($Kr=0.1$), the solid wall acts like an insulated material that results in less thermal dissipation through the wall. Hence, the temperature difference between the solid–fluid interface and cold outer cylindrical surface is moderate, due to which the amount of heat transferred to the fluid region is very minimal (see Fig. 5). For lower thermal conductivity ratio, the isotherms are confined mainly to the solid wall due to the inability of wall thermal conductivity. As *Kr* increases, the isotherms are near stratified state and the flow pattern also changes. For $Kr=10$, the thermal gradient in solid cylinder is very minimal and the solid wall temperature is nearly identical with hot wall temperature due to higher wall thermal conductivity. Further, at higher *Kr*, a strong temperature stratification exists and the buoyant nanofluid motion reveals an enhanced flow strength.

The influence of solid wall thickness on buoyant flow and thermal contours is presented in Fig. 6, by fixing $Ra=10^{6}$, for all three NFs. For $\varepsilon =0.1$, heat is transferred very rapidly from solid cylinder to nanofluid region due to lower thermal resistance of the wall. Thus, heat transfer is mainly dominated by convection, and isotherms are found to be more stratified with strong flow circulation strength. As the wall thickness is increased, the thermal resistance of the wall also increases leading to retardation of heat dissipation through the wall. As a result, the temperature difference between the solid-fluid interface and cold right wall has been reduced, which causes the reduction of flow circulation strength. The thermal stratification, identified from the isotherm contours, also has reasonably disturbed with an increase in inner cylinder thickness. The thermal contours have spread over the solid cylindrical region indicating a significant reduction of thermal gradients in the solid wall as $50 \%$ of annular domain becomes solid $(\varepsilon =0.5)$. As a result, the thermal energy available at the interface may not be sufficient to enhance the fluid flow strength, and can be witnessed from the extreme stream function values.

The variation in buoyant flow and thermal structure due to nanoparticle concentration is discussed in Fig. 7. By increasing the nanoparticle concentration, the thermal conductivity of the nanofluids increase and that leads to an enhancement in the fluid flow intensity. As noticed from streamlines, the flow pattern is not sensitive to the fractional quantity of NPs considered in the study. Further, for lower NP fraction, the flow and thermal structure remains unchanged irrespective of the type of nanoparticle utilized in this investigation. However, a further increase in $\phi$ produces a minor variation of the streamline and isotherm contours.

The thermal dissipation rate from the annular boundary to interface and in turn to the surrounding NFs is highly influenced by various parameters such as *Ra*, *Kr*, $\varepsilon$ and $\phi$. The effect of wide range of these parameters on the global heat dissipation rate at the solid cylinder and interface of the annulus is displayed in Figs. 8, 9, 10, 11, and 12. The effects of *Ra* and *Kr* on overall thermal transport rate at the wall and interface are presented in Fig. 8 for three NFs in comparison with base fluid. In general, the enhancement of thermal dissipation rate strongly depends on the increment in *Ra* caused by increasing the temperature difference or altering the *Kr* value. For all three NFs as well as base fluid, the $\overline{Nu_{w}}$ is higher at low conductivity ratio and lower for larger *Kr*. The thermal dissipation rate at the wall is comparatively higher for base fluid compared to NFs. Though the nanofluid occupied in annular region is not in direct contact with the hot inner wall, the global dissipation rate at solid boundary is slightly influenced by the choice of nanofluid. This can be anticipated from the thermal condition imposed at the interface, which carries the influence of NPs used. On contrast, the heat transport rate at the interface strongly depends on the choice of NP. Further, among the three NPs used in the analysis, Cu-based nanofluid produces enhanced heat dissipation as compared to Al$_2$O$_3$, TiO$_2$ NPs and H$_2$O. In general, the global interface *Nu* enhances with an increment in *Ra* at all conductivity ratios except for $Kr=0.1$, at which $\overline{Nu_{i}}$ remains invariant with *Ra*. For $Kr=0.1$, the wall conductivity is minimum compared to that of nanofluid and hence the solid wall behaves like an insulated material. Therefore, for $Kr=0.1$, the change in temperature difference between the vertical walls has not influenced the global heat transport rate at the interface for all NFs and base fluid considered in this investigation.

Figure 9 exhibits the influence of wall thickness and *Ra* on overall heat transport rate at the wall and interface for the three NFs and base fluid (H$_2$O) with $\phi =0.1$ and $Kr=2$. The impact of wall thickness on thermal dissipation from the wall strongly depends on the *Ra* value. For lower *Ra*, the heat transfer at the wall increases with an increase in wall thickness. However, at higher *Ra*, the heat transfer at the wall declines with an increase in the wall thickness. In fact, the average Nusselt number generally increases with *Ra*. However, due to wall thickness, the enhancement of heat transfer rate with *Ra* has been delayed. As a result, the average *Nu* curves, for different values of $\varepsilon$, coincides at $Ra=10^4$. Also, a sharp increase in $\overline{Nu_w}$ and $\overline{Nu_i}$ with *Ra* can be noticed at lower wall thickness $(\varepsilon =0.1)$ and further increment in $\varepsilon$ reveals minor variation of thermal transport with *Ra*. As discussed earlier, the impacts of different NFs on wall ${\overline{Nu}}$ is indistinguishable, however, base fluid shows a comparatively higher value of $\overline{Nu_{w}}$. As regards to thermal dissipation rate at the interface, $\overline{Nu_{i}}$ enhances with an increase in *Ra* and reducing the wall thickness $(\varepsilon )$ in general. For lower *Ra*, heat transport at the interface does not show any appreciable variation for all three NFs. By increasing *Ra*, the thermal transport rate increases and a marginal increase in the total heat transport rate is observed for Cu–water nanofluid followed by Al$_{2}$O$_3$–water, TiO$_2$–water NFs and H$_2$O for all values of *Ra* and $\varepsilon$. An increase in wall thickness leads to the enhancement of conductive resistance in the solid wall and this results in retardation of the $\overline{Nu_{i}}$.

Figure 10 deals with the effect of *Ra* and $\phi$ on overall heat dissipation rate at the wall and interface for different nanofluids for $Kr=2$ and $\varepsilon =0.2$. An increase in $\phi$ suppresses the overall heat dissipation from the wall, but thermal transport enhancement is observed with an increase in *Ra*. For lower $\phi \le 0.1$, the influence of NPs on $\overline{Nu_{w}}$ is insignificant. However, for NFs with higher $\phi$, minor variation in $\overline{Nu_{w}}$ can be observed for Cu and Al$_2$O$_3$–water NFs contributing to a slightly higher heat transport rate. The overall thermal dissipation rate along the interface has been enhanced as *Ra* and $\phi$ increased. At higher *Ra*, due to enhanced temperature difference, substantial amount of thermal transfer occurs between inner hot and outer cold cylinders. As a result, heat transfer rate at the interface is comparatively higher than at lower *Ra*. A similar prediction has been observed for the three NFs considered in our analysis. Also, on increasing the $\phi$ dispersed in the base fluid, the thermal conductivity of nanofluid is enhanced and with further addition of NPs, the heat removal rate at the interface is further enhanced for all *Ra* values. The estimated global heat transport rate for the NFs with three different types of NPs, such as Cu, Al$_2$O$_3$ and TiO$_2$, reveals that Cu–water nanofluid contributes to better enhancement of heat transport for all values of *Ra* and $\phi$. This may be attributed to the fact that Cu NPs are comparatively more thermal conducting among other chosen NPs. Interestingly, the impact of nanoparticle in enhancing thermal dissipation from interface to adjacent nanofluid is significantly visible at higher *Ra*. The minor change in thermal dissipation from the wall with different NFs could be anticipated from the influence of nanoparticle through the solid–fluid interface. Also, the impact of nanoparticle on $\overline{Nu_{w}}$ is consistent with their influence on thermal dissipation along the interface.

The influence of *Kr* and wall thickness on $\overline{Nu_{w}}$ and $\overline{Nu_{i}}$ are depicted in Fig. 11. An increase in *Kr* or wall thickness produces a sharp decline in the wall heat transfer rate. In general, the thermal dissipation from the wall is a decreasing function of *Kr* and $\varepsilon$. For high $Kr=10$, the influence of wall thickness on thermal transport rate at the wall is insignificant. Also, the impact of different NFs on the heat transport from wall is negligible and H$_2$O produces relatively higher $\overline{Nu_{w}}$ than that of NFs. However, on contrary, the choice of *Kr*, $\varepsilon$ and the type of NFs on thermal dissipation from the interface has reverse impact. The heat dissipation rate at the interface increases sharply for $Kr \le 5$, and moderate enhancement has been noticed for higher *Kr* values. The overall heat transport rate at the interface is an increasing function of *Kr*, but decreases with $\varepsilon$. This is due to the decline in thermal resistance with an increase in *Kr* and decrease in wall thickness. It is noticed that Cu–water provides maximum heat transport compared to other NFs and base fluid. This can be inferred from the fact that higher thermal conductivity of copper clearly influences the overall heat dissipation rate. Interestingly, the change in *Kr* has different influences on the thermal dissipation rate along the wall and interface. The amount of thermal dissipation from the wall is found to be on higher scale as compared to interface for $Kr \le 1$, however, for $Kr > 1$, the interface thermal transport is found to be higher than cylindrical wall.

The influence of $\phi$ and solid wall thickness on overall heat transfer rate at the wall and interface are depicted in Fig. 12. In general, an increase in $\varepsilon$ leads to decrease in ${\overline{Nu}}$ due to enhanced conductive resistance in the wall. An increase in wall thickness leads to retardation of thermal transport to the fluid region. Thus, the average *Nu* at the solid cylinder and interface are decreasing functions of wall thickness. The impact of NFs, pass through the interface, on $\overline{Nu_{w}}$ is profoundly influenced by the thickness of inner cylinder. For thick cylindrical wall, negligible change in thermal dissipation is observed for different NFs. But, a minor variation is noted for the inner cylinder having comparatively lower thickness. This could be explained from the fact that the infiltration influence of nanofluid through the interface is lowered as wall thickness increases. Further, an increase in $\phi$ results in decline of wall heat transport rate for both thick as well as thinner walls. However, at the interface, an increasing trend is observed with an increase in $\phi$ due to enhanced thermal conductivity by the addition of NPs. An in-depth observation from the variation of global *Nu* reveals an important prediction that the change in thermal resistance of the wall, due to variations of wall thickness, has profound influence on thermal dissipation rates along both solid cylinder and interface. The variation in thermal transport rate for different NFs could be vividly noticed along the interface. For all the values of $\phi$ and $\varepsilon$, it is noted that the role of Cu–water in heat transfer enhancement is significant compared to other NFs.

The boundary conditions are chosen in the present analysis in such a way that the interior and exterior cylindrical boundaries are retained at different uniform temperatures, due to which the heat is transferred from inner surface towards outer cylinder. The thermal dissipation rate to nanofluid region greatly rely on the thermal variation along the solid–fluid interface. In addition, these thermal variations depend on various factors, such as the solid wall thickness, *Kr*, *Ra* and $\phi$. Thus, it is important to discuss the temperature changes alongside the interface in order to study the heat dissipation and flow characteristics in the annular region. Figure 13 shows the variation of solid–fluid interface temperature for various magnitudes of wall thickness at $Ra=10^6$, $Kr=2$ and $\phi =0.2$. A similar kind of linear variation in thermal profiles along the interface is observed for thinner $(\varepsilon =0.1)$ and thicker $(\varepsilon =0.5)$ cylinders. Also, the maximum temperature occurs near the upper portion of interface irrespective of wall thickness. This prediction might be from the fact that the clockwise rotating hot fluid rises upwards and hence the interface temperature increases along the wall height. Also, for all wall thickness, the temperature of Al$_{2}$O$_3$ nanofluid is higher compared to the other NFs.

The variation of interface temperature with *Kr* for different NFs at $Ra=10^6$, $\phi =0.2$ and $\varepsilon =0.2$, is presented in Fig. 14. As observed earlier, the temperature at interface increases along the axial direction with maximum temperature is being observed at top region for lower as well as higher *Kr* values. For higher *Kr*, the temperature along interface is found to be on larger scale since higher *Kr* indicates more wall heat conduction, which brings a noticeable increase in the interface temperature. Further, it can be anticipated that the thickness of thermal boundary layer is more pronounced for higher *Kr*. Figure 15a demonstrates the variation in solid–fluid interface temperature at $Ra=10^5$, $Kr=2$ and $\varepsilon =0.2$. For lower $\phi =0.05$, the interface temperature is not altered by type of nanofluid considered. However, a noticeable change in temperature at the interface could be noticed for higher $\phi =0.2$. An increasing trend in the interface temperature is observed in both the cases. The impact of two *Ra* on the interface temperature is depicted in Fig. 15b. The temperature at solid-fluid interface rises along the axial direction for all these NFs. In general, the interface temperature is found to be higher for lower *Ra* in case of Al$_{2}$O$_3$-NFs. Further, the interface temperature moderately increases with axial direction for lower *Ra*, while a steep increase is found for higher *Ra*. For $Kr=2$, since wall conductivity is larger compared to nanofluid, more amount of heat is transferred to the nanofluid domain. Consequently, the temperature at interface declines.

## Conclusions

Conjugate buoyant convective motion in an annular geometry with a solid inner cylinder is numerically analyzed, with examples of the annular region filled with aqueous NFs of Cu, Al$_2$O$_3$ and TiO$_2$ NPs. Based on the vast range of simulations, the novel results can be summarized as follows:

1. The solid wall thickness has a significant effect on regulating flow and temperature distribution inside the annulus. As a result, thermal dissipation at the interface as well as the nanofluid motion declines. It can be concluded that, for varied wall thickness, if $k_w \le k_{nf}$ then ${\overline{Nu}}_{w} > {\overline{Nu}}_{i}$, however, for $k_w > k_{nf}$, the thermal dissipation at interface is found to be higher.
2. An increase in the solid wall to nanofluid thermal conductivity ratio (*Kr*) significantly promotes the heat transport through the wall. Hence, convection is enriched and the thermal transport through the interface is enhanced for higher *Kr*. For given value of $\phi$ and $\varepsilon$, depending on the thermal conductivities of wall and nanofluid, the global Nusselt number is altered. If thermal conductivity of nanofluid is higher compared to solid wall, then the overall heat transport rate at the wall is higher compared to the interface (${\overline{Nu}}_{w} > {\overline{Nu}}_{i}$). The reverse trend has been noticed for the lower nanofluid thermal conductivity.
3. The addition of nano-sized particles to base fluid enhances the thermal dissipation at the solid–fluid interface and percolates the nanofluid fluidity. The total heat transport rate for all three typical NFs enhances with an increase in nanoparticle concentration. For any value of nanoparticle concentration, ${\overline{Nu}}_{w} < {\overline{Nu}}_{i}$ for fixed *Kr* and $\varepsilon$.
4. For given values of *Ra*, $\varepsilon$, *Kr* and $\phi$, the thermal transport rate is significantly higher for Cu–H$_2$O nanofluid compared to other two NFs considered in the present investigation , owing to comparatively higher Cu-thermal conductivity.
  $$\begin{aligned} {\overline{Nu}}(\mathrm{Cu})> {\overline{Nu}}(\mathrm{Al}_2\mathrm{O}_3) > {\overline{Nu}}(\mathrm{TiO}_2) \end{aligned}$$
5. To study the thermal dissipation and flow behavior throughout the domain, the temperature variations along the interface is analyzed. For chosen *Ra*, $\phi$, $\varepsilon$ and *Kr*, the upper portion of interface exhibits higher temperature.
6. For minimal wall thickness, the choice of higher values of *Ra*, *Kr* and $\phi$ enhances the heat transfer through the interface for all the three nanofluids considered in the current analysis.

## List of symbols

*A*: Aspect ratio
$C_p$: Specific heat (J/(kg K))
*d*: Dimensional wall thickness (m)
*D*: Width of annular region (m)
*g*: Acceleration due to gravity (m/s$^2$)
*H*: Height of the annular enclosure (m)
*k*: Thermal conductivity [W/(m K)]
*Kr*: Thermal conductivity ratio
*Nu*: Local Nusselt number
${\overline{Nu}}$: Average Nusselt number
*p*: Dimensional fluid pressure (Pa)
*P*: Dimensionless fluid pressure
$$\vec{q}$$: Velocity vector
*Pr*: Prandtl number
*Ra*: Rayleigh number
$r_i$: Radius of inner cylinder (m)
$r_o$: Radius of outer cylinder (m)
*t*: Dimensionless time
$$t^*$$: Dimensional time (s)
*T*: Dimensionless temperature
(*r*, *z*): Dimensional radial and axial co-ordinates (m)
(*R*, *Z*): Dimensionless radial and axial co-ordinates
(*u*, *v*): Dimensional velocity components in (*r*, *z*) direction (m/s)
(*U*, *V*): Dimensionless velocity components in (*R*, *Z*) direction

## Greek symbols

$$\alpha$$: Thermal diffusivity (m$$^2$$/s)
$\beta$: Thermal expansion coefficient (1/K)
$\varepsilon$: Dimensionless wall thickness
$$\zeta$$: Dimensionless vorticity
$$\theta$$: Dimensional temperature (K)
$$\lambda$$: Radius ratio
$\mu$: Dynamic viscosity (kg/(m s))
$$\nu$$: Kinematic viscosity (m$$^2$$/s)
$\rho$: Fluid density (kg/m$^3$)
$\phi$: Volume fraction of nanoparticle
$\psi$: Dimensionless stream function

## Subscripts

*c*: Cold
*f*: Base fluid
*h*: Hot
*i*: Interface
*nf*: Nanofluid
*p*: Nanoparticle
*w*: Wall
